# Luteal phase estradiol versus luteal phase GnRH antagonist administration: their effects on antral follicular size coordination and basal hormonal levels

**Published:** 2011

**Authors:** Batool Rashidi, Roya Nasiri, Haleh Rahmanpour, Ensieh Shahrokh Tehraninejad, Maryam Deldar

**Affiliations:** 1 Valie-Asr Reproductive Health Research Center, Faculty of Medicine, Tehran University of Medical Sciences, Tehran, Iran.; 2 Department of Obstetrics and Gynecology, Aria Hospital, Faculty of Medicine, Islamic Azad University of Medical Sciences, Mashhad Branch, Mashhad, Iran.; 3 Department of Obstetrics and Gynecology, Faculty of Medicine, Zanjan University of Medical Sciences, Zanjan, Iran.

**Keywords:** *Follicular synchronization*, *Estradiol*, *GnRH antagonist*

## Abstract

**Background:**

The differential efficacy between long GnRH agonist with antagonist can partly be due to the preexisting differences in the early antral follicles before ovarian stimulation.

**Objective:**

To compare the effect of pretreatment by estradiol with GnRH antagonist on antral follicular size coordination and basal hormone levels in GNRH antagonist protocol.

**Materials and Methods:**

On cycle day 3 (control/day 3), women underwent measurements of early antral follicles by ultrasound and serum FSH and ovarian hormones then were randomized to receive oral estradiol 4mg/day (*n*=15) or 3mg cetrorelix acetate (*n*=15) in luteal phase before subsequent antagonist protocol. Participants were re-evaluated as on control/day 3.

**Results:**

There was a significant reduction of mean follicular sizes in each group after medical intervention (7.63±2.11 Vs. 4.30±0.92 in group A and 8.73±1.96 Vs. 4.13±1.11 in group B) (p=0.0001). The magnitude of follicular size reduction was significantly higher in group B (-4.60±2.04 Vs. -3.33±2.28) (0.027). There was a non significant attenuation of follicular size discrepancies in two groups. FSH and inhibin B levels in the day 3 of the next cycle in both groups were significantly decreased but did not have significant difference between two groups.

**Conclusion:**

Both luteal E2 and premenstrual GnRH antagonist administration reduces the follicular sizes significantly and GnRH antagonist acts more potently than E2 in this way but attenuation of follicular size discrepancies in both treatment is not significant.

## Introduction

During the early follicular phase of menstrual cycle inconsistent sensitivity of early antral follicles to FSH and their dissimilar sizes play an important role in the establishment of the follicular dominance (1, 2). In controlled ovarian hyper stimulation (COH), asynchronous follicular growth is counterproductive and reduces the number of mature oocytes and embryos and the likelihood of conception (3).

Long GnRH agonist protocol is clinically applied for synchronous and coordinate growth of early antral follicles and prevention of endogenous LH surge, during COH for ART. The initial flare- up and the rather long period until pituitary suppression and hypoestrogenic side effects are the disadvantages of long GnRH agonist protocol (4-6). The differential efficacy between long GnRH agonist with antagonist and short protocols can partly be due to the preexisting differences in the early antral follicles before ovarian stimulation (7).

Size discrepancy in early antral follicles can lead to marked follicular size discrepancies at the end of COH and smaller number of mature oocytes and embryos (8). It has been demonstrated that lowering endogenous gradient FSH secretion during the late luteal phase by administering either estradiol or GnRH antagonist can result in more synchronized antral follicles in the next early follicular phase (9) and achievement of adequate coordination of multiple follicular growths to trigger ovulation is one of the key objectives of COH (10).

In the present study we wished to compare the effectiveness of late luteal phase administration of Estradiol valerate with GnRH antagonist in reducing antral follicular diameters and disparities, and FSH and inhibin B levels on day 3 of the next cycle. 

## Materials and methods

This interventional single blind clinical trial was conducted on 30 volunteers, attending the university based infertility clinic. All the subjects were 20-35 years old women with male factor infertility and candidate for ICSI who met the following inclusion criteria: No current or past systemic disease, Regular menstrual cycles (25-35 days) and Body mass index (BMI) ranging 18-27 kg/m2. 

Those with history of hormone therapy for the past preceding 3 months or single ovary were excluded. Written informed consent was obtained and the study protocol was approved by Ethics committee of Tehran University of Medical Sciences. Participants were allocated into two groups employing permuted block randomization method and followed for two consecutive menstrual cycles (60 study cycles). 

On day 3 of their first menstrual cycles (baseline/day 3), transvaginal ultrasound scans of ovaries and blood sampling for serum E2, FSH, inhibin B measurement were performed. Ultrasound scans (US) were performed with a 3.5 to 5 MHZ multifrequency vaginal probe (AGUILA, Esaote, Italy) by a single operator who was not aware of the treatment schedule. In US examination the number and size of the 2-12 mm follicles (mean of two orthogonal diameters) and the diameters of ovaries were recorded. Ovarian volume was calculated as height in width in depth of the ovary multiplied by 0.526. Intra-analysis coefficients of variation (CVs) for follicular and ovarian measurements were <5% and their lower limit of detection were 0.1 mm. Serum FSH was determined by chemiluinescence technique (Diarson, Italy). Intra-assay and inter-assay CV were respectively 3% and 5% and lower limit detection was 0.1 mI u/ml). Serum inhibin B was determined by double-antibody ELISA (Sertec, Varilhes, France) lower limit detection was 10 Pg/ml, and intra-assay and inter assay CV were<6% and <9% respectively. Serum E2 was determined by chemiluinescence technique (Diarson, Italy). Lower limit detection was 14 Pg/ml, and intra- assay and inter assay CV were 8% and 9% respectively.

In group A (n=15), 4mg/day oral tablets of Estradiol valerate (Estradiol, Abooreihan, Iran) was administered from day 20 of the same cycle until day 2 of their next cycle for suppression of endogenous FSH secretion. Subjects in group B (n=15) received a single subcutaneous injection of 3 mg GnRH antagonist (Cetrolelix acetate, cetrotide, Serono lab, Boulogne, France) on day 25 of the same cycle. Similar US and laboratory examination were done on day 3 of their next cycles, as in their preceding cycles were performed (Estradiol/day 3 or GnRH antagonist/day 3) then these two groups enrolled the antagonist protocol.

**Statistical analysis**

Measure of central tendency used was the mean and measure of variability was standard deviation (SD) and coefficient of variation. To evaluate the magnitude of follicular size discrepancies from baseline/day 3 to E2/day 3 or GnRH antagonist/day 3, we used Fisher exact test. In addition, SD/mean ratios for follicular size were calculated. A p-value of <0.05 was considered statistically significant.

## Results

The E2 group and the GnRH antagonist group contain 15 women in each group that were comparable with regard to age (27.1±3.2 Vs. 27.0±3.3 years), body mass index (25.4±1.7 Vs. 26.2±2.2 kg/m^2^) and duration of infertility (4.9±1.7 Vs. 5.4±2.4 years). Follicular and ovarian measurements and hormonal results are summarized in table I. There was a significant reduction of mean follicular sizes in each group after medical intervention (p=0.0001). The magnitude of follicular size reduction was significantly higher in group B (-4.60±2.04 Vs. -3.33±2.28) (p=0.027) (Table II). 

There was a non-significant attenuation of follicular size discrepancies in E2/day3 and GnRH antagonist/day3, as shown by SD/mean ratio of follicular sizes in table I. In comparison with GnRH antagonist, E2 resulted in better follicular size homogeneity. 

FSH and inhibin B levels on day 3 of the next cycle in both groups were significantly decreased but did not have difference between two groups. Serum E2 level significantly increased after treatment in group A, but did not change in group B.

**Table I:** Ultrasound and hormonal results during two consecutive menstrual cycles in women receiving E2 or GnRH antagonist in the luteal phase.

**GnRH antagonist group (B)/Day 3**			**E2 group(A)/Day 3**			**Parameter**
**p-value**	**GnRH antagonist**	**Baseline**	**p value**	**E2**	**Baseline**	
0.648**	9.60±1.57	9.73±1.31	0.515**	10.17±1.23	10.33±1.35	No. of follicles (range)
0.0001**	4.13±1.11	8.73±1.96	0.0001**	4.30±0.92	7.63±2.11	Mean follicular size (mm)
0.246***	1.11	1.96	0.869***	0.92	2.11	SD follicular size (mm)
0.206*	9.67±2.03	9.35±1.83	0.407*	9.5±1.44	9.33±1.65	Mean ovarian volume (cm3)
0.0001*	21.36±13.18	30.73±11.06	0.0001*	19.46±11.38	29.53±9.27	Serum inhibin B (pg/mL)
0.246*	45.20±17.81	50.80±19.11	0.001*	75.07±34.97	48.53±22.64	Serum E2 (pg/mL)
0.002*	5.53±1.60	6.49±1.66	0.025*	4.96±2.03	5.77±1.83	Serum FSH (mIU/mL)

P<0.05 =Significant level.

* Paired samples t-test .

** Wilcoxon signed ranks .

*** Levene's test for equality of variances.

**Table II:** Comparison of pre and post treatment ultrasound and hormonal result differences between group A and B.

**p-value**	**Differences between pre and post treatment cycles**		**Parameter**
	**GnRH antagonist group (B)**	**E**_2_ ** group (A)**	
0.932	-0.13±1.6	-0.17±1.42	No. of follicles (range)
0.027	-4.60±2.04	-3.33±2.28	Mean follicular size (mm)
0.619	+0.33±1.39	+0.17±1.09	Mean ovarian volume (cm3)
0.696	-9.37±4.88	-10.07±4.84	Serum inhibin B (pg/mL)
0.0001	-5.60±17.90	+26.53±23.04	Serum E2 (pg/mL)
0.720	-0.96±0.95	-0.81±1.25	Serum FSH (mIU/mL)

Repeated measurement analysis of variance .

P<0.05 =Significant level.

## Discussion

This study showed that luteal FSH suppression by either E2 or GnRH antagonist administration reduces the size and improves the homogeneity of early antral follicles during the early follicular phase. These results were in accordance of Fanchines *et al* studies but in his studies each treatment was compared separately with no intervention (8, 9). 

Compared with GnRH agonist long protocols, the introduction of GnRH antagonist protocols for controlled ovarian hyper stimulation has offered the great opportunity to reduce the duration of treatment, the consumption of gonadotrophins and to lower the physical and psychological burden for patients submitted to pituitary desensitization (11). While in short GnRH agonist and GnRH antagonist regimens, the slight reduction in the number of retrieved oocytes and in the pregnancy rate reported has been partly attributed to the absence of synchronization of the follicular cohort before ovarian stimulation as compared with long GnRH agonist (6, 7). 

For that reason, more attention has been paid to the potential interest of steroid pre-treatments to program cycles, to modify the hormonal environment in relation to the negative feedback exerted by steroids on endogenous gonadotrophin secretion and therefore to synchronize the follicular cohort before stimulation. Indeed, both oral contraceptive pill (OCP), synthetic progestogens and Estradiol have been largely used for many years to program cycles (11).

Oral contraceptives, due to their potent anti-FSH action, may exert similar, or even stronger, coordinating effects on early follicular development compared with luteal E2 or premenstrual GnRH antagonist administration (12). 

In recent Cochrane review it was stated, that combined OCP pre-treatment in GnRH antagonist cycles is associated with fewer clinical pregnancies and more days and a higher amount of gonadotrophin therapy and progestogen pre-treatment in GnRH agonist cycles, is associated with more clinical pregnancies and fewer ovarian cysts, at last, in estrogen pre-treated GnRH antagonist cycles, compared to no pre-treatment, more oocytes are retrieved but a higher amount of gonadotrophin therapy is needed (13). 

In contrast, Fanchin *et al* showed that synchronization of the follicular cohort with estrogen pre-treatment, followed by a short wash-out period before starting stimulation, was associated with an increase in the number of follicles and oocytes retrieved (14). 

In a recent study the addition of GnRH antagonist to luteal E2 for luteal suppression before ovarian stimulation for IVF does not improve IVF outcomes in poor responders (16). The reduction of antral follicle size discrepancy in our study was not significant and this may be due to small sample size.

## Conclusion

In conclusion, these data show both luteal E2 and premenstrual GnRH antagonist administration reduces the follicular sizes significantly but whether strong homogenization of the follicular cohort by GnRH antagonist or estrogen pre-treatment has differential effects on oocyte yield, endometrial quality and COH outcome remains to be determined in an adequately powered prospective study.

## Note

***Registration ID in IRCT: IRCT201103052568N2***
